# Optimizing weighting functions for cryo-electron microscopy

**DOI:** 10.52601/bpr.2021.210001

**Published:** 2021-04-30

**Authors:** Jing Cheng, Xinzheng Zhang

**Affiliations:** 1 National Laboratory of Biomacromolecules, CAS Center for Excellence in Biomacromolecules, Institute of Biophysics, Chinese Academy of Sciences, Beijing 100101, China; 2 University of Chinese Academy of Sciences, Beijing 100049, China; 3 Center for Biological Imaging, CAS Center for Excellence in Biomacromolecules, Institute of Biophysics, Chinese Academy of Sciences, Beijing 100101, China

**Keywords:** Cryo-electron microscopy (Cryo-EM), Cross correlation coefficient (CCC), Weighting function

## Abstract

The frequency-dependent signal to noise ratio of cryo-electron microscopy data varies dramatically with the frequency and with the type of the data. During different steps of data processing, data with distinct SNR are used for calculations. Thus, specific weighting function based on the particular SNR should be designed to optimize the corresponding calculation. Here, we deduced these weighting functions by maximizing the signal to noise ratio of cross correlated coefficients. Some of our weighting functions for refinement resemble that used in the existing software packages. However, weighting functions we deduced for motion correction, particle picking and the refinement with overlapping densities differ from those employed by existing programs. Our new weighting functions may improve the calculation in these steps.

## INTRODUCTION

During the imaging of cryo-electron microscopy (cryo-EM) biological samples with a transmission electron microscope (TEM), proteins may undergo irreversible radiation damage (Baker and Rubinstein 2010). To minimize protein damage, only a limited number of electrons are therefore permitted during imaging. A total dose of ~40 e^−^/Å^2^ is typical for a cryo-EM image, a dose that is much lower than ~2,000 e^−^/Å^2^ used in conventional HRTEM (high resolution TEM) imaging. Low-dose imaging however produces images with low signal-to-noise ratios (SNRs) that become a major problem in subsequent cryo-EM data processing steps.

Most of the electrons scattered by an atom scatter through small angles and thereby contribute to the low-frequency signals in TEM images. In cryo-EM images, scattered electrons from proteins produce low-frequency signals that are stronger than the high-frequency signals. Additionally, during imaging, the envelope function of the contrast transfer function (CTF) further decreases the signals exponentially with increasing frequency (De Jong and Van Dyck 1993). In contrast, the shot noise in a cryo-EM image acquired in the electron counting mode may be treated approximately as white noise, of which the power spectrum remains constant for all frequencies. Thus, the frequency-dependent SNR of a cryo-EM image decreases rapidly with increasing frequency.

In different cryo-EM data processing stages, such as motion correction, particle selection, and alignments of 2D images with a reference, cross correlation coefficients (CCCs) are usually calculated to compare two images. An accurate calculation of the CCCs has to take the frequency-dependence of SNRs into account. For instance, data with low SNRs should be multiplied by a weighting lower than those with high SNRs. Programs such as Frealign and Jalign use CTF and SNR-dominated weighting to calculate the CCCs during refinement (Grigorieff 2007; Sun *et al*. 2020). The maximum likelihood method also uses data-driven SNR-dependent weighting to optimize the likelihood (Scheres 2012). However, in different stages of data processing, cryo-EM data exhibit distinct SNRs. For particle selection, the signal of the protein is dominant because only low-frequency signals are involved. For motion correction, the shot noise is dominant because each frame is generated by doses of only 1–2 e^−^/Å^2^. In some more complicated instances, such as image alignments against a sub-volume (focused refinement or block-based reconstruction), other protein densities apart from that of the target protein are involved in the calculation of CCCs. These protein densities can be treated as noise but this changes the SNRs. Thus, different weighting functions need to be developed and used in the different stages of data processing. Calculating or optimizing the frequency-dependent weighting functions at different stages of cryo-EM data processing remains unresolved.

Here, we estimate the spectral SNR (SSNR) of a cryo-EM image and derive different weighting functions according to the different types of SSNR ($\gg$1, ≈1, and $\ll $1) by optimizing the SNR of the CCCs. Depending on the type of SNR of the data, the application of corresponding weighting functions may improve motion correction, particle selection, and alignment.

## RESULTS AND DISCUSSION

### The SSNR of a cryo-EM image

**Figure 1:**
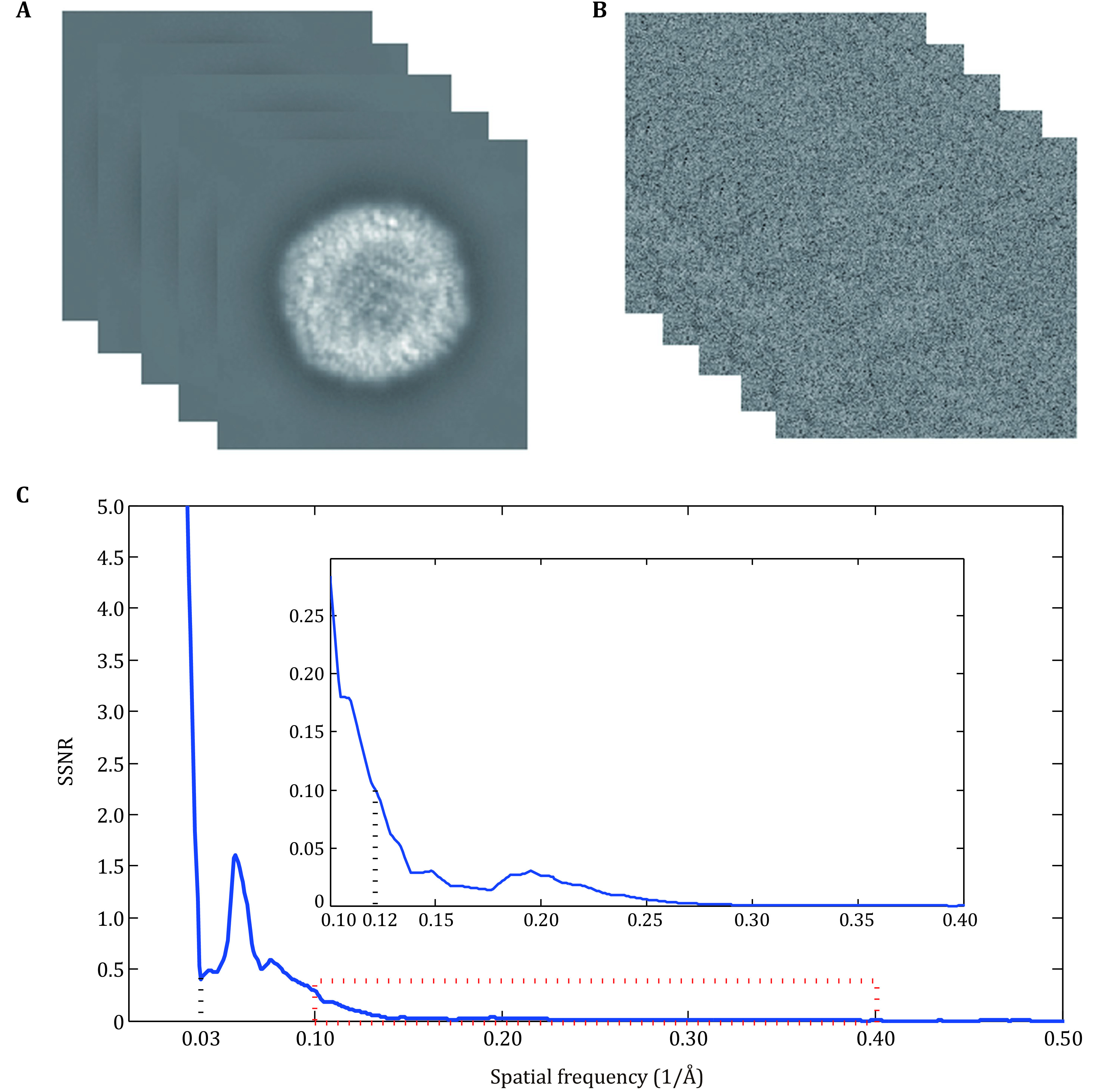
SSNR varies greatly with frequency. **A** 2D projections free of noise. **B** 2D raw images. **C** The SSNR value calculated by FRCs. SSNR is particularly high until frequency 0.03, and drops rapidly at this point. The areas with very small SSNRs are marked by a red dashed box and enlarged above

A typical SSNR of a protein in a cryo-EM image (Fig. 1A and B) was estimated from apoferritin data (see the section of Methods). The SSNR (Fig. 1C) is much larger than 1 at spatial frequencies ranging from 0 to 0.03 Å^−1^, and decreases rapidly to ~1 at frequencies ranging from 0.03 to 0.12 Å^−1^. A further decrease in signal leads to a SNR below 0.1 at frequencies larger than 0.12 Å^−1^.

Because a motion-corrected micrograph is averaged from a set of dose-fractioned frames (usually 30–50 frames), an $N$-fold dose accumulation enhances the SNR by factor $\sqrt N $ (Lee *et al*. 2014). Hence, the SSNR of a single frame may be evaluated by dividing the SSNR of the averaged micrograph by $\sqrt {{N_{{\rm{frames}}}}} $. Therefore, the SNR of a single frame is much lower than that of the averaged micrograph.

We partitioned the frequency range into three intervals of resolution depending on the value of the frequency-dependent SSNR, specifically (1) the low-resolution interval in which the SSNR is larger than 10, (2) the medium-resolution interval in which the SSNR is between 0.1 and 10, and (3) the high-resolution interval in which the SSNR is below 0.1. The assumption that SSNR $\gg 1$ for the high-resolution interval and SSNR $\ll 1 $ for the high-resolution interval is used in the derivations of formulae below.

### Weighting in motion correction

The ice-embedded proteins move during imaging as a consequence of either the irradiation of the electron beam (Brilot *et al*. 2012) or residues in stage movements. Such movements of the protein degrade the quality of the cryo-EM image. To correct for the motion of proteins, the direct electron detector records proteins in movie mode, a series of frames that record the movement of several proteins. Using motion correction software (Timothy Grant and Grigorieff 2015; Li *et al*. 2013; Zheng *et al*. 2017), the trajectory of proteins from frame-to-frame can be estimated and compensated before the averaging of all frames. Currently, the calculation of the movement of proteins in different software depends on the CCCs between frames or the CCCs between a frame and the averaged image without applying any CTF oscillation weights.

The distinguishing characteristic of the SSNR of a frame is that the assumption SNR $\ll 1$ holds in most regions.

Weighted CCCs between frames are expressed as

1 $c{c_w}^{{\rm{frame}} \cdot {\rm{frame}}} = \sum\nolimits_k {W(k) \cdot {X_1}(k) \cdot {X_2}^ * (k)}, $

with

2 ${X_1}(k) = F(k) \cdot CTF(k) + {N_1}(k),$

3 ${X_2}(k) = F(k) \cdot CTF(k) + {N_2}(k),$

with $W(k)$ denoting the weighting function, $F(k)$ the protein structure factor, $CTF(k)$ the CTF oscillation with envelop damping, "$* $" the conjugate operation, ${N_1}(k)$ and ${N_2}(k)$ the shot noise of two frames, respectively. Substituting Eqs. 2 and 3 into 1 gives

4 \begin{equation*}\begin{split} 
c{c_w}^{{\rm{frame}} \cdot {\rm{frame}}} =\; &\sum\nolimits_k W(k) \cdot [F(k) \cdot CTF(k) + {N_1}(k)]  \cdot \\& [ \left({F(k) \cdot CTF(k)} \right) + {N_2}(k)]  ^ * .
\end{split}\end{equation*}

We derive the weighting function by maximizing the SNR of the CCCs, specifically,

5 $\frac{{dSN{R_{cc_w^{{\rm{frame}} \cdot {\rm{frame}}}}}}}{{dW}} = 0,$

with

6 \begin{equation*}\begin{split} 
SNR =& {{\left[ \displaystyle \sum\nolimits_k W(k) \cdot CT{F^2}(k) \cdot {F^2}(k)\right] ^2 }}/\\&
\displaystyle \sum\nolimits_k \left\{{{[ W(k) \cdot F(k) \cdot CTF(k) \cdot {N_2}^ * (k)] }^2} \right.\\&
+ {{[ W(k) \cdot {F^ * }(k) \cdot CTF(k) \cdot {N_1}(k)] }^2}\\&\left.
+ {{[ W(k) \cdot {N_1}(k) \cdot {N_2}^ * (k)] }^2}\right\} 
\end{split}\end{equation*}

giving

7 $W(k) = \frac{{{F^2}(k) \cdot CT{F^2}(k)}}{{{F^2}(k) \cdot CT{F^2}(k) \cdot [ N_1^2(k) + N_2^2(k)]  + N_1^2(k) \cdot N_2^2(k)}}.$

When processing a single frame, we assume that the noise intensity ${N^2}$ is far greater than the signal intensity ${F^2}$. With this assumption, we obtain

8 $W(k) = \frac{{{F^2}(k) \cdot CT{F^2}(k)}}{{N_1^2(k) \cdot N_2^2(k)}}.$

Because the data are collected in counting mode, noise at different frequencies is normalized, and hence this weighting function simplifies to

9 $W(k) = {F^2}(k) \cdot CT{F^2}(k).$

When the motion is estimated by calculating the CCCs between a frame and the averaged image, the expression becomes

10 \begin{equation*}\begin{split} 
c{c_w}^{{\rm{frame \cdot frame}}} = \;&\sum\nolimits_k W(k) \cdot [F(k) \cdot CTF(k) + N(k)] \cdot \\& [ F(k) \cdot CTF(k) + N'(k)]  ^ * ,
\end{split}\end{equation*}

with $N(k)$ denoting the noise of the frame, and $N'(k)$ the noise of the average of frames,

11 $\frac{{dSN{R_{cc_w^{{\rm{frame}} \cdot {\rm{mgraph}}}}}}}{{dW}} = 0\;.$

Here, we have ${N^2} \gg {F^2},{N^2} \gg {N'^2}$, and hence the weighting function becomes

12 $W(k) = \frac{{{F^2}(k) \cdot CT{F^2}(k)}}{{{{N'}^2}(k) + {F^2}(k) \cdot CT{F^2}(k)}}.$

**Figure 2:**
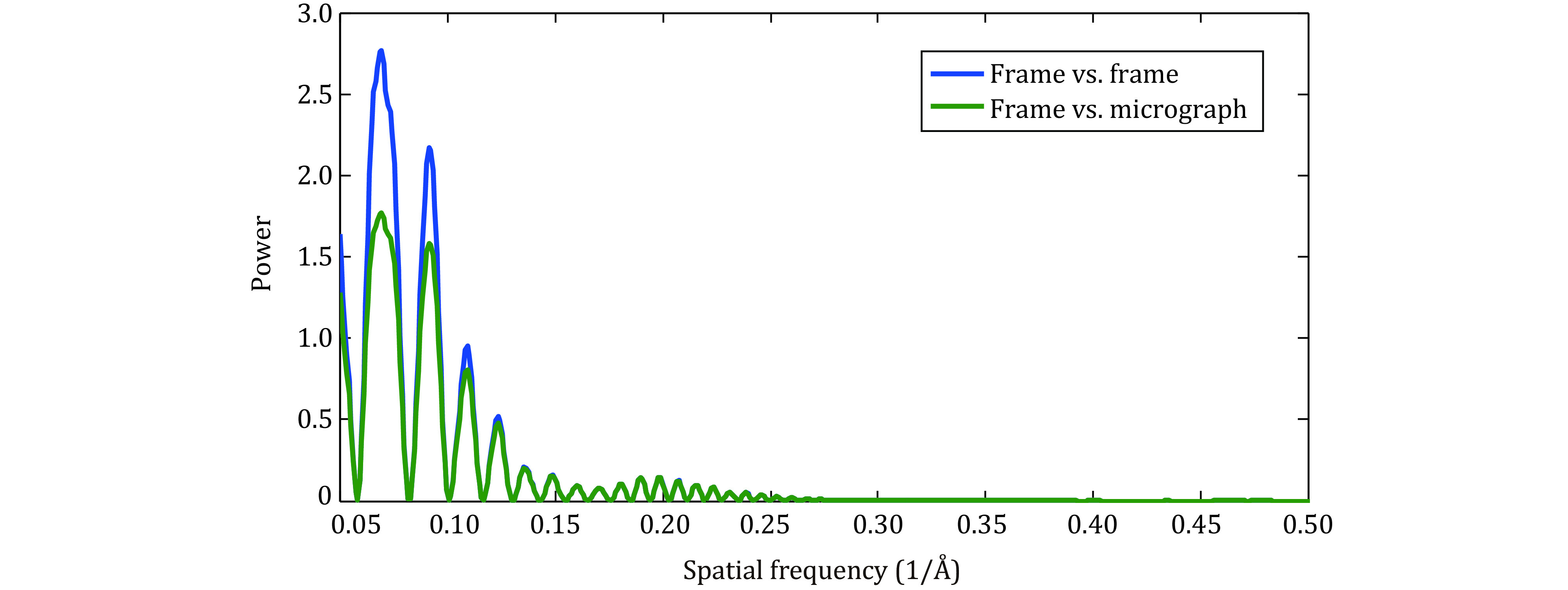
Power spectrum of a frame after being applied with weighting function in two different cases of the motion correction: calculating similarity between frames (navy) and similarity between a frame and averaged micrograph (olive)

Currently, during the preprocessing of cryo-EM micrographs, motion correction is performed before the calculation of the CTF parameters. Therefore, the weighting function used for motion correction only contains a term related to the envelope function (Li *et al*. 2013; Zheng *et al*. 2017;Timothy Grant and Grigorieff 2015). We propose that an extra weighting term related to the CTF oscillations (Fig. 2) should be applied to obtain a better performance in motion correction after preprocessing.

### Weighting in particle selection

The high-resolution reconstruction of protein structures by a single-particle analysis often requires a selection of particles from micrographs. Template-based particle selection with CCC is widely used in single-particle analysis (Chen and Grigorieff 2007; Hall and Patwardhan 2004; Huang and Penczek 2004), which usually involves only low-frequency data (<30 Å^−1^). Thus, we assume SNR $\gg $ 1 for particle selection. We calculated the optimized weighting by maximizing SNR of the CCCs,

13 $cc_w^{{\rm{pick}}} = \sum\nolimits_k {W(k) \cdot M(k) \cdot {T^ * }(k)} \;.$

Here, $M$(*k*) denotes the micrograph, which contains particles in various views and noise, and $T$ the noise-free low-resolution template. The expression for *M*(*k*) is

14 \begin{equation*}\begin{split} 
M(k) =\;& {F_1}(k) \cdot CTF(k) + {F_2}(k) \cdot CTF(k) \\& +  \cdots  + {F_n}(k) \cdot CTF(k) + N(k)\;,
\end{split}\end{equation*}

15 $T(k) = {F_1}(k)\;,$

with ${F_1}$ pertaining to the first particle present in the micrograph, and ${F_n}$ the *n*-th particle, and hence the CCC becomes

16 \begin{equation*}\begin{split} 
cc_w^{{\rm{pick}}} = \;&\sum\nolimits_k W(k) \cdot \left[ {F_1}(k) \cdot CTF(k) +  \cdots  + {F_n}(k) \right.\cdot\\&\left. CTF(k) + N(k) \right] \cdot {F_1}^ * (k)\;.
\end{split}\end{equation*}

By applying condition $\dfrac{{dSN{R_{cc_w^{{\rm{pick}}}}}}}{{dW}} = 0$, we obtain

17 $W(k) = \frac{{CTF(k)}}{{CT{F^2}(k) \cdot F_2^2(k) +  \cdots  + CT{F^2}(k) \cdot F_n^2(k) + {N^2}(k)}}.$

**Figure 3:**
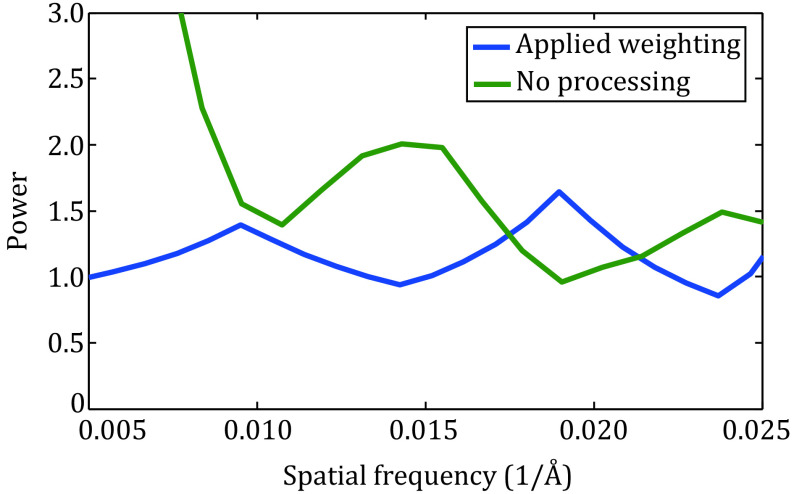
Power spectrum of image applied with weighting of particle picking (navy) and power spectrum of original image (olive). The horizontal coordinate retains only the frequencies involved in particle picking

Because SNR $\gg $ 1, we have ${F^2}(k) \gg {N^2}(k)$ and the raw micrograph may be represented by *CTF*(*k*)·*F*(*k*). The final optimized weighting approximates a whitening of the power spectrum of the micrograph (Fig. 3). Therefore, we propose that a whitening filter should be applied to both micrographs and reference to achieve a better particle selection.

### Weighting in refinement

Accurate refinement is important in achieving high-resolution structures. Currently, refinements usually comprise two searches, a global search with coarse step size compared against a medium resolution model and a local search near a set of specific parameters with fine step size compared against an improved high-resolution model. In local searches, the signal of the 2D images at medium resolutions is not sensitive enough because the 2D references are similar at this resolution. Thus, the signal of a 2D image of high resolution for which SNR $\ll $ 1 plays an important role in determining the angular and translational parameters. This assumption does not hold when the reconstruction has only a medium resolution.

In a typical single-particle reconstruction, extracted particles from cryo-EM images usually only contain the target protein itself. However, for a focused refinement without subtracting surrounding densities or block-based reconstruction, unexpected densities associated with overlapping proteins intrude into the calculation rather than densities of the target protein. Assuming that these densities are not correlated to the model used for calculating CCCs, we simply treat the densities as noise in the derivation of formulae, specifically,

18 $cc_w^{{\rm{temp}} \cdot {\rm{image}}} = \sum\nolimits_k {W(k) \cdot X(k) \cdot {T^ * }(k)}\;,$

with

19 $X(k) = {F_1}(k) \cdot CTF(k) + {F_2}(k) \cdot CTF(k) + N(k)\;,$

20 $T(k) = {F_1}(k) + N'(k)\;,$

and ${F_1}$ denoting the signal from the target protein, and ${F_2}$ the signal from other proteins. Noise in the raw image $N$ is far greater than noise of the template $N'$. Hence, with this approximation, $W(k)$ is deduced by maximizing the SNR of $cc_w^{{\rm{temp}} \cdot {\rm{image}}}$ yielding

21 $W(k) = \frac{{CTF(k) \cdot FSC(k)}}{{CT{F^2}(k) \cdot F_2^2(k) + {N^2}(k)}}.$

Because ${F_2}$ equals 0 for a typical single-particle refinement, therefore, $W(k)$ is

22 $W(k) = \frac{{CTF(k) \cdot FSC(k)}}{{{N^2}(k)}}.$

**Figure 4:**
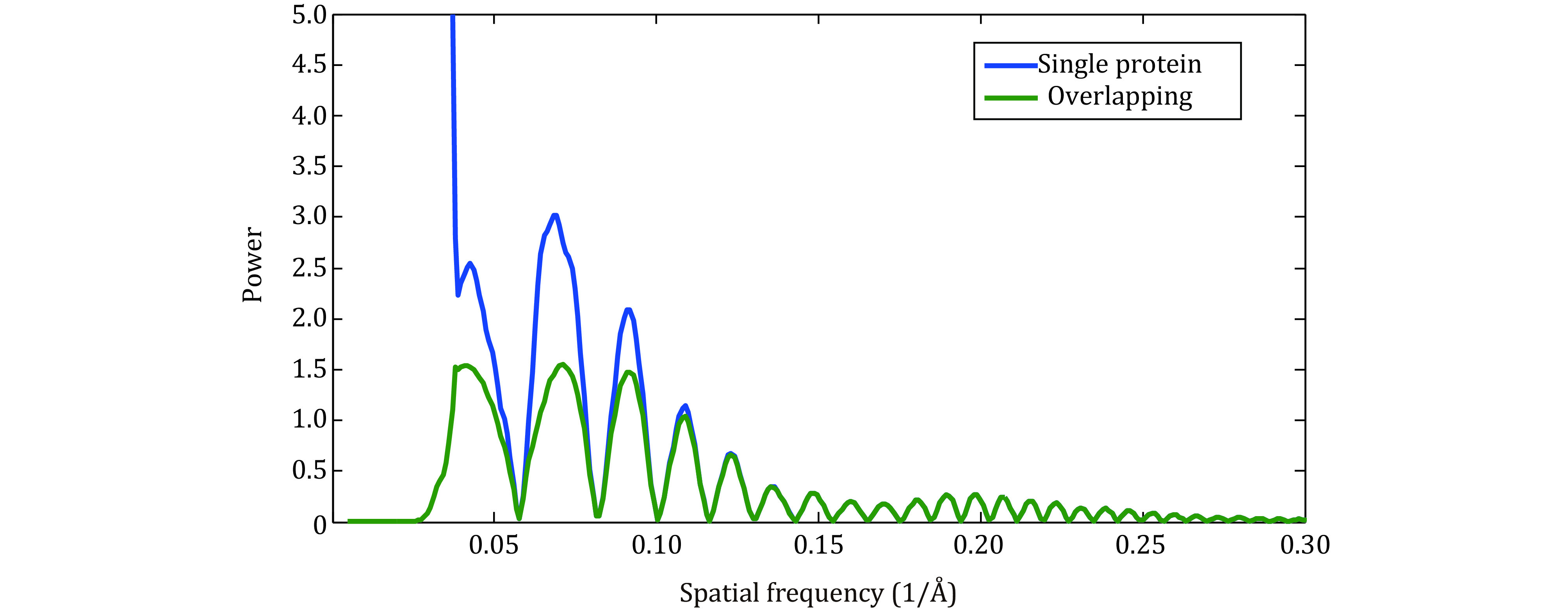
Power spectrum of image applied with weightings deduced for refinement: olive for overlapping case and navy for single protein case. Here we consider the sub-volume subjected to focused refinement is 1/3 of the remaining part, thus the overlapping density is set to three times the target protein density

A similar weighting function has been used in the program Jalign (Sun *et al*. 2020), in defocus refinements (Su *et al*. 2017; Zivanov *et al*. 2018), and in the program Cistem (Grant *et al*. 2018). With high-resolution refinements, $F_2^2$ is much smaller than ${N^2}$, and therefore the weighting function for particles with overlapping densities becomes the same as for the typical case (Fig. 4).

Thus, for local refinements, which lead to high-resolution reconstructions, overlapping densities do not change the weighting function. However, if the reconstruction is limited to a medium resolution, we believe that refinements will benefit with the application of the new weighting function.

## CONCLUSION

We have analyzed the decay of structure factors of proteins with frequency in cryo-EM, and then divided the frequencies into three distinct intervals depending on the SNR value: the low-resolution interval, which appears in particle selection, with SSNR $\gg $ 1; the medium-resolution interval with SSNR ≈ 1, which holds for medium-resolution alignments; and the high-resolution interval for high-resolution alignments for which SSNR $\ll $ 1. For motion correction, SSNR $\ll $ 1 holds in most regions. The different stages of cryo-EM data processing correspond to different frequency ranges, and by calculating the maximum SNR for the CCCs, we derived optimized weighting functions for various processing stages. We believe that our optimized weighting functions may improve cryo-EM data processing in some stages.

## METHODS

### Estimation of SSNRs of cryo-EM images

To study the variation of the frequency-dependent SNR in a cryo-EM image, an accurate calculation of the SSNR is necessary. The SSNR of an image is estimated from the Fourier ring correlation (FRC) between two independent aligned data sets (Sindelar and Grigorieff 2012),

23 $S S N R(k) \approx {f_{{\rm{mask2D}}}}\frac{{{N_k}}}{{\sum\nolimits_{i,j} {\sum\nolimits_{n = 1}^{{N_{{\rm{images}}}}} {{{\left| {CT{F_n}(i,j)} \right|}^2}} } }} \cdot \frac{{2FRC(k)}}{{1 - FRC(k)}}\;,$

where *FRC*(*k*) denotes the 2D analog of the Fourier shell correlation, ${N_k}$ the number of Fourier pixels contained in the frequency ring, $f_{{\rm{mask2D}}}$ the mean-squared value of the soft edged mask, *i* and *j* index the location of the grid component in Fourier space, and the black dot "·" signifies the dot-product operation for the corresponding points on the ring of Fourier pixels.

We calculated the FRC between projections of the two half cryo-EM reconstruction of apoferritin. The images have a defocus variation from 0.4 to 2.1 μm to remove the CTF oscillations. After converting the FRC to SSNR using Eq. 23, we achieved the final estimate of the SSNR.

## Conflict of interest

Jing Cheng and Xinzheng Zhang declare that they have no conflict of interest.
